# Supplementary material for: MetaRibo-Seq measures translation in microbiomes
Source: Nat Commun. 2020 Jun 29;11:3268. doi: 10.1038/s41467-020-17081-z (PMC7324362; doi:10.1038/s41467-020-17081-z)
Supplement: Supplementary file 10 — Supplementary Data 7 [file 41467_2020_17081_MOESM10_ESM.zip › File2/Confidence_VeryHigh_Taxonomy/89009_out.krona.html]

Javascript must be enabled to view this page.

members
magnitude
magnitudeUnassigned
count
unassigned
taxon
rank

89009\_out

7

2
7
superkingdom

976
7
phylum

200643
7
class

171549
7
order

171552
family
7

7

SRS017701\_contig\_number\_3647SRS019910\_contig\_number\_16498SRS048164\_contig\_number\_12799SRS077392\_contig\_number\_contig-100\_1473.237322SRS1041033\_contig\_number\_11958SRS1041133\_contig\_number\_11865SRS145497\_contig\_number\_11139
species
2049047
